# Supplementary material for: Balanced imitation sustains song culture in zebra finches
Source: Nat Commun. 2021 May 7;12:2562. doi: 10.1038/s41467-021-22852-3 (PMC8105409; doi:10.1038/s41467-021-22852-3)
Supplement: Supplementary file 1 — Supplementary Information [file 41467_2021_22852_MOESM1_ESM.pdf]

Balanced imitation sustains song culture in zebra finches

Ofer Tchernichovski, Sophie Eisenberg-Edidin & Erich D. Jarvis

Supplementary Information

Supplementary Note 1. Testing for imitation from neighbor tutor songs

We tested if pupil's who imitated their tutor's song poorly were influenced by the song of other tutors in their room, whom they could hear. We did not record the entire acoustic environments that existed in each room in each developmental period. However, we found 22 clutches with pupils who imitated poorly (lowest quartile), where we have recordings from at least one neighboring tutor that raised chicks in the same room at the same time. In cases of more than a single neighbor tutor, we selected the tutor that was imitated most accurately by his sons. We then measured the similarity between the song of pupils who imitated their tutor poorly, and the song of that 'popular' neighbor tutor. We performed this analysis in four different colony rooms. We compared these similarities to shuffled data: pairing each pupil with a non-neighbor tutor from a different room. We found that the mean similarity within a room was not higher than that across rooms (within a room: 38.7±2.6%; across rooms: 40.1±3.6%). Although we cannot exclude the possibility that these pupils might have copied song elements from tutors that we did not record, results are consistent with earlier observations, suggesting that zebra finches mainly imitate tutors that live with them and, in those conditions, ignore songs from birds they cannot interact with.

Supplementary Note 2. Mixed effects statistical models

2.1 Linear mixed-effects model for syllable diversity

```
Linear mixed-effects model fit by ML

Model information:
   Number of observations            81
   Fixed effects coefficients         2
   Random effects coefficients      116
   Covariance parameters              3

Formula:  Influence ~ 1 + DivTutor + (1 | tutorID) + (1 | pupilID)

Model fit statistics:
   AIC       BIC       LogLikelihood    Deviance
   703.18    715.15    -346.59          693.18

Fixed effects coefficients (95% CIs):
   Name                 Estimate    SE        tStat      DF    pValue      Lower      Upper
   {'(Intercept)'}      44.968      25.974    1.7313     79    0.087302    -6.7311    96.668
   {'DivTutor'   }      8.8155      9.9074    0.88979    79    0.37628     -10.905    28.536
```

2.2 Linear mixed-effects model for song similarity (tutor->pupil):

Model information:
Number of observations 160
Fixed effects coefficients 2
Random effects coefficients 228
Covariance parameters 3

Formula:
Similarity ~ 1 + tutorinfo + (1 | TutorID) + (1 | PupilID)

Model fit statistics:

AIC	BIC	LogLikelihood	Deviance
1368.9	1384.2	-679.44	1358.9

Fixed effects coefficients (95% CIs):

Name	Estimate	SE	tStat	DF	pValue	Lower	Upper
{'(Intercept)'}	4.5151	34.44	0.1311	158	0.89586	-63.506	72.537
{'tutorinfo' }	21.614	11.077	1.9512	158	0.052801	-0.2646	43.492

Random effects covariance parameters (95% CIs):
Group: TutorID (68 Levels)

Name1	Name2	Type	Estimate	Lower	Upper
{'(Intercept)'}	{'(Intercept)'}	{'std'}	11.765	8.5868	16.12

Group: PupilID (160 Levels)

Name1	Name2	Type	Estimate	Lower	Upper
{'(Intercept)'}	{'(Intercept)'}	{'std'}	9.5709	3.432e-225	2.6691e+226

Group: Error

Name	Estimate	Lower	Upper
{'Res Std'}	10.301	2.5171e-194	4.2158e+195

2.3 Linear mixed-effects model for song influence (pupil->tutor):

Linear mixed-effects model fit by ML

Model information:
Number of observations 160
Fixed effects coefficients 2
Random effects coefficients 228
Covariance parameters 3

Formula:
Influence ~ 1 + tutorSongDiversiy + (1 | TutorID) + (1 | PupilID)

Model fit statistics:

AIC	BIC	LogLikelihood	Deviance
1378.7	1394.1	-684.34	1368.7

Fixed effects coefficients (95% CIs):

Name	Estimate	SE	tStat	DF	pValue	Lower	Upper
{'(Intercept)'}	-87.206	33.012	-2.6417	158	0.0090794	-152.41	-22.005

{ tutorSongDiversiy }	50.645	10.623	4.7672	158	4.2092e-06	29.662	71.627

Random effects covariance parameters (95% CIs):
Group: TutorID (68 Levels)

Name1	Name2	Type	Estimate	Lower	Upper
{'(Intercept)'}	{'(Intercept)'}	{'std'}	10.52	7.3672	15.021

Group: PupilID (160 Levels)

Name1	Name2	Type	Estimate	Lower
{'(Intercept)'}	{'(Intercept)'}	{'std'}	10.687	NaN

2.4.2 Linear mixed-effects model for pupil vs tutor song diversity

Model information:

Number of observations	160
Fixed effects coefficients	2
Random effects coefficients	228
Covariance parameters	3

Formula:
PupilInfo ~ 1 + tutorinfo + (1 | TutorID) + (1 | PupilID)

Model fit statistics:

AIC	BIC	LogLikelihood	Deviance
-148.1	-132.72	79.05	-158.1

Fixed effects coefficients (95% CIs):

Name	Estimate	SE	tStat	DF	pValue	Lower	Upper
{'(Intercept)'}	1.8714	0.21987	8.5116	158	1.2535e-14	1.4372	2.3057
{'tutorinfo' }	0.39667	0.070882	5.5961	158	9.4591e-08	0.25667	0.53666

Random effects covariance parameters (95% CIs):
Group: TutorID (68 Levels)

Name1	Name2	Type	Estimate	Lower	Upper
{'(Intercept)'}	{'(Intercept)'}	{'std'}	0.043436	0.019049	0.099045

Group: PupilID (160 Levels)

Name1	Name2	Type	Estimate	Lower	Upper
{'(Intercept)'}	{'(Intercept)'}	{'std'}	0.11119	NaN	NaN

Group: Error

Name	Estimate	Lower	Upper
{'Res Std'}	0.088047	NaN	NaN

2.4.3 Linear mixed-effects model for pupil song diversity (all factors combined):

Formula:
PupilSongDiversity ~ 1 + Similarity + Influence + TutorSongDiversity + (1 | TutorID) + (1 | PupilID)

Model fit statistics:

AIC	BIC	LogLikelihood	Deviance
-167.68	-146.15	90.839	-181.68

Fixed effects coefficients (95% CIs):

Name	Estimate	SE	tStat	DF	pValue	Lower	Upper
{'(Intercept)'}	1.8762	0.21598	8.687	156	4.7489e-15	1.4496	2.3028
{'Similarity' }	0.0032593	0.00069207	4.7095	156	5.4504e-06	0.0018923	0.0046263
{'Influence' }	-0.00034424	0.00068015	-0.50613	156	0.61348	0.18298	0.47618
{ TutorSongDiversity }	0.32958	0.074217	4.4408	156	1.6891e-05	-0.0016877	0.00099925

Random effects covariance parameters (95% CIs):

Group: TutorID (68 Levels)

Name1	Name2	Type	Estimate	Lower	Upper
{'(Intercept)'}	{'(Intercept)'}	{'std'}	0.041927	0.018252	0.096313

Group: PupilID (160 Levels)

Name1	Name2	Type	Estimate	Lower	Upper
{'(Intercept)'}	{'(Intercept)'}	{'std'}	0.10243	NaN	NaN

Group: Error

Name	Estimate	Lower	Upper
{'Res Std'}	0.082244	NaN	NaN

Supplementary figures

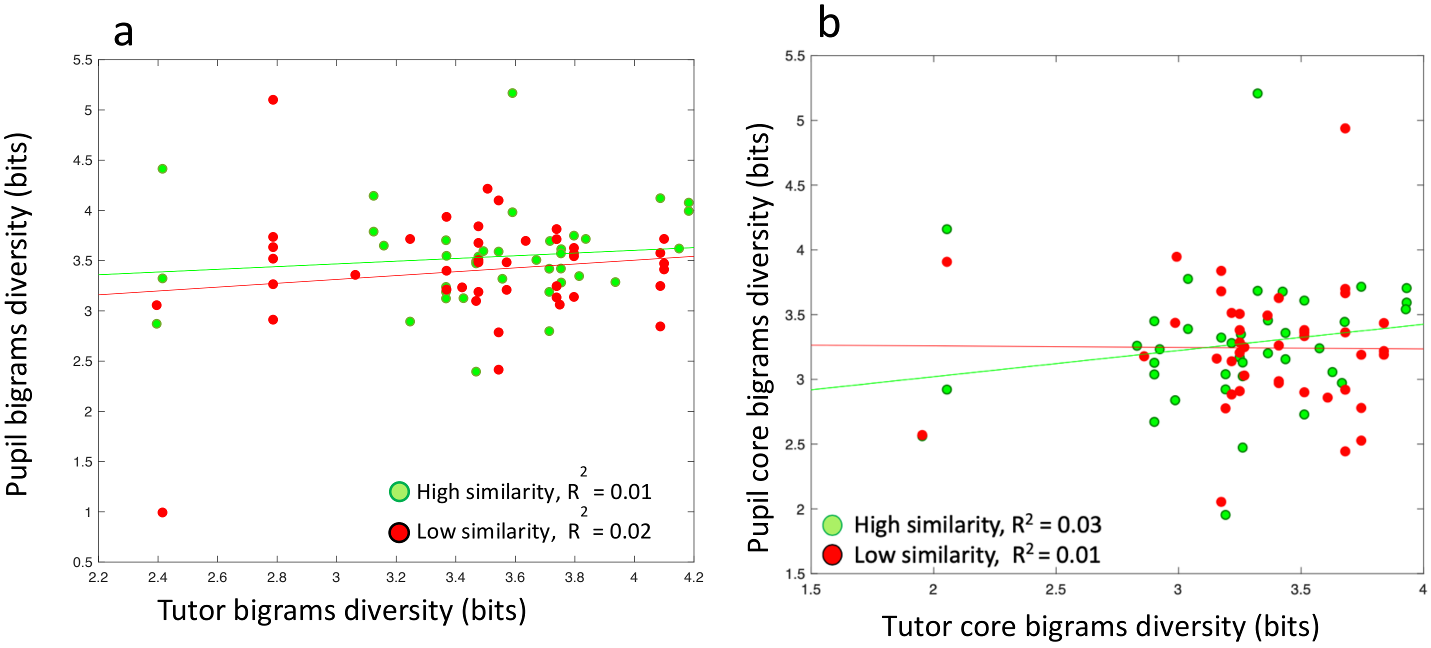

Supplementary Fig. 1: **a** Tutor vs. pupil diversities of syllable type transitions (bigrams). **b** same as (a) for core-motif syllable types transitions are uncorrelated. Core motif syllables were identified as syllable types that repeat in a persistent serial order in each song motif, excluding introductory notes and repeated calls. Source data for this figure is in Supplementary Data File 1.

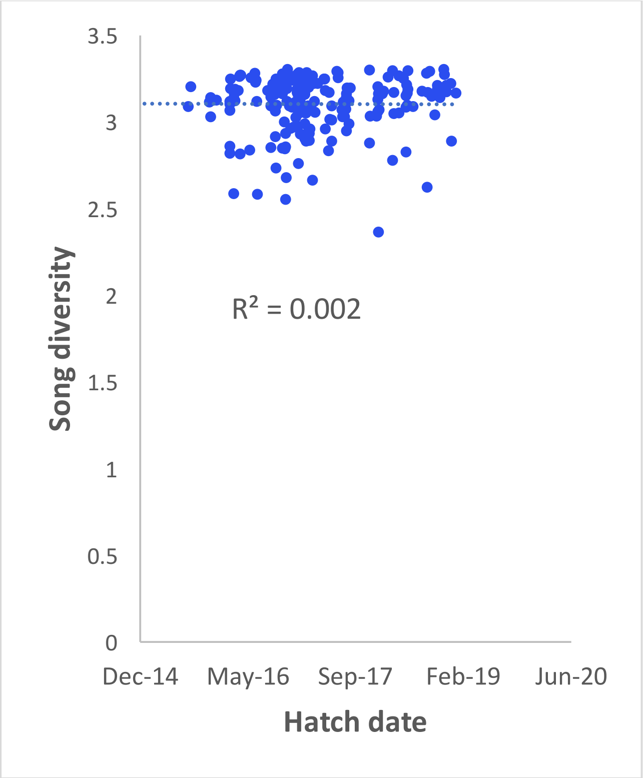

Supplementary Fig. 2: Time course of song diversity during the lifetime of the colony. Source data for this figure is in Supplementary Data File 1
