# Supplementary material for: Balanced imitation sustains song culture in zebra finches
Source: Nat Commun. 2021 May 7;12:2562. doi: 10.1038/s41467-021-22852-3 (PMC8105409; doi:10.1038/s41467-021-22852-3)
Supplement: Supplementary file 3 — Reporting Summary [file 41467_2021_22852_MOESM3_ESM.pdf]

nature research

Corresponding author(s): Ofer Tchernichovski and Erich D Jarvis

Last updated by author(s): Mar 6, 2021

Reporting Summary

Nature Research wishes to improve the reproducibility of the work that we publish. This form provides structure for consistency and transparency in reporting. For further information on Nature Research policies, see our Editorial Policies and the Editorial Policy Checklist.

Statistics

For all statistical analyses, confirm that the following items are present in the figure legend, table legend, main text, or Methods section.

n/a	Confirmed	
☐	☒	The exact sample size (*n*) for each experimental group/condition, given as a discrete number and unit of measurement
☐	☒	A statement on whether measurements were taken from distinct samples or whether the same sample was measured repeatedly
☐	☒	The statistical test(s) used AND whether they are one- or two-sided<br>*Only common tests should be described solely by name; describe more complex techniques in the Methods section.*
☐	☒	A description of all covariates tested
☐	☒	A description of any assumptions or corrections, such as tests of normality and adjustment for multiple comparisons
☐	☒	A full description of the statistical parameters including central tendency (e.g. means) or other basic estimates (e.g. regression coefficient) AND variation (e.g. standard deviation) or associated estimates of uncertainty (e.g. confidence intervals)
☐	☒	For null hypothesis testing, the test statistic (e.g. *F*, *t*, *r*) with confidence intervals, effect sizes, degrees of freedom and *P* value noted<br>*Give P values as exact values whenever suitable.*
☒	☐	For Bayesian analysis, information on the choice of priors and Markov chain Monte Carlo settings
☒	☐	For hierarchical and complex designs, identification of the appropriate level for tests and full reporting of outcomes
☒	☐	Estimates of effect sizes (e.g. Cohen's *d*, Pearson's *r*), indicating how they were calculated

Our web collection on statistics for biologists contains articles on many of the points above.

Software and code

Policy information about availability of computer code

Data collection	All data was collected using our open source software Sound Analysis Pro (SAP) version 2018.02 (http://soundanalysispro.com/).
Data analysis	All the statistical data analysis was performed using Matlab version is 2020B 9.9.0 (https://www.mathworks.com/products/matlab.html). All custom scripts are in a Github link: https://github.com/oferon/Balanced-Imitation

For manuscripts utilizing custom algorithms or software that are central to the research but not yet described in published literature, software must be made available to editors and reviewers. We strongly encourage code deposition in a community repository (e.g. GitHub). See the Nature Research guidelines for submitting code & software for further information.

Data

Policy information about availability of data

All manuscripts must include a data availability statement. This statement should provide the following information, where applicable:

- Accession codes, unique identifiers, or web links for publicly available datasets
- A list of figures that have associated raw data
- A description of any restrictions on data availability

All audio recordings of the song library we collected and analyzed for this study are in a Github link: https://github.com/oferon/Balanced-Imitation.
Further, our entire zebra finch song library (.wav files), family trees of all individuals, example sonograms, bird IDs, and more have been deposited in a public Dropbox folder: https://www.dropbox.com/sh/vvrz3o2inb1ynxk/AAAI6oaJ_pkrML8ON_kQa-_xa?dl=0

Field-specific reporting

Please select the one below that is the best fit for your research. If you are not sure, read the appropriate sections before making your selection.

☐ Life sciences ☒ Behavioural & social sciences ☐ Ecological, evolutionary & environmental sciences

For a reference copy of the document with all sections, see nature.com/documents/nr-reporting-summary-flat.pdf

Behavioural & social sciences study design

All studies must disclose on these points even when the disclosure is negative.

Study description	Behavioral quantative song data were collected, using automated sound recording software (SAP)
Research sample	Our zebra finches came from a population of approximately 800 birds at the Rockefeller University Field Research Center.
Sampling strategy	Our sample size was 160 male-female zebra finch pairs and their offspring, 228 birds total, recorded in 15 cohorts. The main criterion to be included in the study was that the male pupils of the parents needed to be raised with the parents for 90 days until adulthood, in a single cage without others families in the cage. We attempted to try to sample all breeding cages, and thus no sample size minimum calculations were deemed necessary. As far as we are aware this is the largest sample size of this type of analyses in a songbird.
Data collection	All male song data were recorded by Ofer Tchernichovski (OT) using Sound Analysis Pro Recording software at 16bit 44.1kHz. All recordings were done by OT, and analyses by OT and Sophie Eisenberg-Edidin, blind to experimental groups. Recording hardware includes M-Audio 2424 8 channel sound card and omnidirectional AudioTechnica Microphones.
Timing	All songs were recorded between August 2018 and July 2019. The exact dates and time for each song are available in our song database. https://www.dropbox.com/sh/vvrz3o2inb1ynxk/AAAI6oaJ_pkrML8ON_kQa-_xa?dl=0
Data exclusions	None
Non-participation	There was no non-participation
Randomization	Randomization was not necessary, as we analyzed song imitation in the entire dataset available to us. This includes all cross-fostered individuals. This includes all 30 cross fostered individuals in our colony that was necessary as part of the breeding program.

Reporting for specific materials, systems and methods

We require information from authors about some types of materials, experimental systems and methods used in many studies. Here, indicate whether each material, system or method listed is relevant to your study. If you are not sure if a list item applies to your research, read the appropriate section before selecting a response.

Materials & experimental systems

n/a	Involved in the study	
☒	☐	Antibodies
☒	☐	Eukaryotic cell lines
☒	☐	Palaeontology and archaeology
☐	☒	Animals and other organisms
☒	☐	Human research participants
☒	☐	Clinical data
☒	☐	Dual use research of concern

Methods

n/a	Involved in the study	
☒	☐	ChIP-seq
☒	☐	Flow cytometry
☒	☐	MRI-based neuroimaging

Animals and other organisms

Policy information about studies involving animals; ARRIVE guidelines recommended for reporting animal research

Laboratory animals	Adult (> 90 days old) male and female breeding pairs, and their male offspring were used. Dates of birth and recording dates are available in our song library.
Wild animals	No wild animals were used in this study.
Field-collected samples	No field collected samples were used in this study.
Ethics oversight	The Rockefeller University

Note that full information on the approval of the study protocol must also be provided in the manuscript.
